# A Sec-dependent effector, CLIBASIA_04425, contributes to virulence in ‘*Candidatus* Liberibater asiaticus’

**DOI:** 10.3389/fpls.2023.1224736

**Published:** 2023-07-24

**Authors:** Shushe Zhang, Xuefeng Wang, Jun He, Song Zhang, Tingchang Zhao, Shimin Fu, Changyong Zhou

**Affiliations:** ^1^ Citrus Research Institute, Southwest University/National Citrus Engineering Research Center, Chongqing, China; ^2^ State Key Laboratory for Biology of Plant Diseases and Insect Pests, Chinese Academy of Agriculture Sciences, Institute of Plant Protection, Beijing, China; ^3^ Guangxi Citrus Breeding and Cultivation Engineering Technology Center Academy of Specialty Crops, Guangxi, Guilin, China

**Keywords:** Citrus Huanglongbing (HLB), *C*Las, effector, *C*Las4425, cell death, virulence factor

## Abstract

Citrus Huanglongbing (HLB) is the most destructive citrus disease worldwide, mainly caused by ‘*Candidatus* Liberibacter asiaticus’ (*C*Las). It encodes a large number of Sec-dependent effectors that contribute to HLB progression. In this study, an elicitor triggering ROS burst and cell death in *Nicotiana benthamiana*, CLIBASIA_04425 (*C*Las4425), was identified. Of particular interest, its cell death-inducing activity is associated with its subcellular localization and the cytoplasmic receptor *Botrytis*-induced kinase 1 (BIK1). Compared with *C*Las infected psyllids, *C*Las4425 showed higher expression level in planta. The transient expression of *CLas4425* in *N*. *benthamiana* and its overexpression in *Citrus sinensis* enhanced plant susceptibility to *Pseudomonas syringae* pv. *tomato* DC3000 Δ*hopQ1-1* and *C*Las, respectively. Furthermore, the salicylic acid (SA) level along with the expression of genes *NPR1*/*EDS1*/*NDR1*/*PR*s in SA signal transduction was repressed in *C*Las4425 transgenic citrus plants. Taken together, *C*Las4425 is a virulence factor that promotes *C*Las proliferation, likely by interfering with SA-mediated plant immunity. The results obtained facilitate our understanding of *C*Las pathogenesis.

## Introduction

Citrus Huanglongbing (HLB), the most devastating disease of citrus, has been known in East Asia for over a century (Reinking, 1919; Lin, 1956). Diseased trees have leaves with a blotchy mottle, and the branches gradually die as the disease progresses (Martinelli and Dandekar, 2017). It has occurred in ca. 50 countries throughout the world and over 300 counties of 10 provinces in the mainland of China (Zhou, 2020). Even though the fight against HLB has never stopped in China, the disease still causes tremendous losses to the citrus industry. Since HLB arrived in 2005, citrus production in Florida has decreased by 74% (Singerman and Rogers, 2020). The causal agents of HLB are three *Candidatus* Liberibacter species bacteria, named for their geographic distributions: ‘*Ca.* Liberibacter asiaticus’ (*C*Las), ‘*Ca*. Liberibacter africanus’ (*C*Laf), and ‘*Ca*. Liberibacter americanus’ (*C*Lam) (Bové, 2006). Realizing the disease severity, researchers have made tremendous efforts to uncover the pathosystem of the HLB causal agents. However, their obligate and phloem-limited characteristics hamper mechanistic studies of the pathogenicity (Perilla-Henao and Casteel, 2016). Among three Liberibacter species, *C*Las is the most prevalent strain and thus is commonly studied. Exploring the battle strategies between *C*Las and citrus plants are critical for the establishment of control remedies.

A ‘zig-zag’ model has been used to clarifiy the plant-pathogen interaction (Jones and Dangl, 2006). In this theory, pathogens generally utilize pathogen-associated molecular patterns (PAMPs) and effectors to attack the host. *C*Las has been observed to stimulate non-self-recognition immune responses *in planta*, including phloem blockage, increased levels of salicylic acid, and production of reactive oxygen species (ROS) (Wang and Trivedi, 2013). Nevertheless, a molecular understanding of the process remains undiscovered. Suppression or avoidance of plant immunity is necessary for successful pathogen colonization. Transmitted by *Diaphorina citri* Kuwayama (ACP) into phloem elements, *C*Las evades recognition by plant outer membrane receptors. Effectors serve as an important component of pathogens, playing critical roles in pathogenesis. Lacking common type III and IV secretion systems (T3SS and T4SS), *C*Las delivers effectors *via* general secretion pathway (Sec), T1SS, and noncanonical secretion pathway (Andrade et al., 2020). Among them, Sec-dependent effectors are mostly investigated (Prasad et al., 2016). Transient expression of *C*Las effectors in *Nicotiana benthamiana* is used for large-scale screening of virulence factors, and many are identified to affect plant innate immunity (Pitino et al., 2016; Du P. et al., 2021).

Hypersensitive response (HR)-based cell death is a common consequence of pathogen recognition by the plant immune system. To date, CLIBASIA_05315 (SDE1), CLIBASIA_05150, and AGH17470 have been shown to stimulate HR, including ROS burst and programmed cell death (Pitino et al., 2016; Ying et al., 2019; Du et al., 2022). Interacting with the papain-like cysteine proteases (PLCPs), SDE1 contributes to HLB progression *via* attenuating PLCPs activity (Clark et al., 2018; Clark et al., 2020). By contrast, many effectors are determined to block HR induced by two common elicitors, pro-apoptotic mouse protein BCL2-associated X protein (BAX) and *Phytophthora infestans* elicitin inverted formin 1 (INF1) (Zhang et al., 2019; Pang et al., 2020). SDE15 suppression of plant immunity is dependent on the citrus protein accelerated cell death 2 (ACD2). Moreover, CLIBASIA_05330 can even suppress SDE1-triggered HR (Shen et al., 2022). Intriguingly, the virulence of CLIBASIA_00460 relies on temperature, which affects its pathogenicity *via* interfering with subcellular localization (Liu et al., 2019).

Here, we investigate a *C*Las Sec-dependent effector CLIBASIA_04425 (*C*Las4425) that can also trigger cell death by transient expression in *N*. *benthamiana*. Additionally, subcellular localization and the cytoplasmic receptor *Botrytis*-induced kinase 1 (BIK1) are indispensable for its cell death-inducing activity. *C*Las4425-expressing *N*. *benthamiana* and citrus plants are more vulnerable to pathogen infection. Molecular evidence showed *C*Las4425 impairs the salicylic acid (SA) signaling pathway. Accordingly, we conclude that *C*Las4425 is a virulence effector of *C*Las and can be perceived by the plant immune system.

## Materials and methods

### Plants, bacteria, and growth conditions

The seedlings of *N*. *benthamiana* were grown for 5-6 weeks at 25°C in a greenhouse with 18 h light/6 h darkness. The citrus plants were grown at 28°C. *C*Las-infected sugar orange plants (*Citrus reticulata*) were used for DNA extraction, as well as a source for graft-inoculation. The epicotyl segments of Wanjincheng (*C*. *sinesis*) were used for gene transformations. *Pseudomonas syringae* pv. *tomato* DC3000 (DC3000) Δ*hopQ1-1* was grown on King’s B (KB) medium.

### Nucleotide extraction and detection analysis

For *C*Las detection, total DNA was extracted from midrib sections of sugar orange plants using Biospin Omini Plant Genomic DNA Extraction Kit (BioFlux, Hangzhou, China). The *C*Las detection was conducted by both PCR and quantitative PCR (qPCR). The PCR was performed using 16S rDNA primer OI1/OI2c with 2X Taq Master Mix (Vazyme, Nanjing, China) in a 20 μL reaction system. The bacterial populations (*C*Las cells μg^-1^ of citrus DNA) were quantified with qPCR assay that was described by Du M. et al. (2021). In the qPCR assay, *CLasgyrA* of *C*Las was detected, and *18S rRNA* of citrus was used as the internal reference. BlastTaq™ 2X qPCR MasterMix (abm, Canada) was used for qPCR amplification. Primer pairs are listed in **Table S1**.

Total RNA was extracted from plant tissues and psyllids using RNAiso Plus (Takara, Japan). In a 20-μL volume, 1μg of total RNA was reverse transcribed with All-In-One 5X RT MasterMix (abm) following the manufacturer’s instructions. Quantitative RT-PCR (qRT-PCR) assays were conducted to analyze gene transcript level. Primer pairs of selected genes are listed in **Table S1**. The genes *NbACTIN* and *CsGAPDH* were used as endogenous controls. The qRT-PCR analyses were performed in a 10 μL reaction system. The 2^-ΔΔCt^ method was used for the determination of relative gene transcription (Livak and Schmittgen, 2001).

### Plasmid constructs and preparation

Primer pairs and vectors used are listed in **Table S1**. Fragments were amplified by PCR by using Q5 high-fidelity DNA polymerase and digested with appropriated restriction endonucleases (NEB, Peking, China), followed by ligation into vectors using ClonExpress II One Step Cloning Kit (Vazyme). For phoA assay, the fragment of *C*Las4425 signal peptide (4425SP) was inserted into pET-mphoA (*T7* promoter). For the transient expression assay, the coding sequences were cloned into the binary Potato virus X (PVX) vector pGR107. To construct the overexpression vector, *CLas4425* was ligated into pLGN (*35S* promoter). For the VIGS assay, fragments amplified from *N*. *benthamiana* cDNA were cloned into the binary vector pTRV2. All constructs were sequenced by Tsingke (Chongqing, China).

### Alkaline phosphatase assay

The resulting vector pET-4425SP-mphoA, pET-phoA (positive control), and pET-mphoA (negative control) were transformed into *Escherichia coli* Rosetta-gami2 (DE3), then the *E*. *coli* cells were incubated at 37°C overnight in indicator LB solid medium [90 ng/mL 5-bromo-4-chloro-3-indolyl phosphate (BCIP), 0.1 mM isopropyl-β-D-thiogalactopyranoside (IPTG), 75 μM Na_2_HPO_4,_ and 50 μg/mL Kanamycin]. The blue transformants indicated that fusion proteins were secreted outside the cells, while white colonies demonstrated a lack of phoA activity.

### *Agrobacterium tumefaciens*-mediated transient expression

*A. tumefaciens* GV3101 (pJIC SA_Rep) strains harboring the respective constructs were cultured in LB medium supplemented with the appropriate antibiotics at 28°C, and then harvested and suspended in infiltration buffer [10 mM 2-(*N*-morpholine)-ethane sulfonic acid (MES), 10 mM MgCl_2_, 0.2 mM acetosyringone to pH 5.6] to appropriate concentrations (OD_600_ of 0.2 for INF1, BAX, otherwise OD_600_ of 0.6 was used). After dark incubation for 2 h at 28°C, the *A*. *tumefaciens* suspensions were infiltrated into the upper leaves of 6-leaf-old *N*. *benthamiana* plants with needless syringes. Cell death was observed at 5-8 dpi. For the gene expression analysis, 0.1 mg of samples was detached at 2, 4, 6, and 8 dpi, respectively. The collected samples were quickly frozen in liquid nitrogen and stored in -80°C.

### 3, 3’-diaminobenzidine and trypan blue staining

H_2_O_2_ accumulation was detected using DAB staining. The *N. benthamiana* leaves were detached at 3 dpi and incubated in DAB solution (1 mg/mL DAB, 1:2000 Tween-20, and 10 mM Na_2_HPO_4_), then vacuumed until the leaves were infiltrated with DAB solution (Du et al., 2022). The sample leaves were placed on a shaker at 60 rpm overnight, then distained in 95% ethanol and photographed.

For programmed cell death (PCD) observation, infiltrated leaves were incubated in trypan blue staining solution (0.067% w/v trypam blue, 11.11% w/v phenol, 1 volume of glycerol, lactic acid and distilled water, respectively, and 6 volume of 95% ethanol), boiled for 2 min, then transferred into chloral hydrate (1.25 g/mL). The sample leaves were places on a shaker at 60 rpm until they were fully destained (Shen et al., 2022).

### Subcellular localization of *C*Las4425 in plant cells

The mature form of *C*Las4425 fused with red fluorescent protein (RFP) was cloned into PVX. For its entrance into the nuclei, the nuclear localization signal (NLS) was added at N-terminus of *C*Las4425:RFP. Leaves of *N. benthamiana* were collected at 3 dpi. Subcellular localization of *C*Las4425 was determined using an FV3000 confocal equipped with a UV light source (Olympus, Tokyo, Japan). H2B:green fluorescent protein (GFP) and Pm : GFP were used as markers for the nucleus and cytoplasmic membrane, respectively.

### Virus-induced gene silencing assay in *N*. *benthamiana*

The TRV-based gene silencing system was conducted to silence *BAK1*, *SOBIR1*, and *BIK1* in *N. benthamiana*. *A*. *tumefaciens* GV3101 strains carrying respective pTRV2 constructs were mixed with pTRV1 in equal ratios to a final OD_600_ of 0.2. pTRV2:*GUS* served as a control, and pTRV2:*PDS* was used to visualize the silencing process (Liu et al., 2002). *A*. *tumefaciens* suspensions were infiltrated into cotyledons of 4-leaf-old seedlings. The silencing efficiency was validated with qRT-PCR of RNA from the leaves in the locations corresponding to the albino leaves.

### Immunoblotting

Protein was extracted from infiltrated leaves using the Plant Protein Extraction Kit (Solarbio, Peking, China). Total protein was separated by 12% SDS-PAGE, and the protein samples were transferred to a polyvinlidene difluoride (PVDF) membrane. Anti-HA-tag served as primary monoclonal antibody at a dilution of 1:2000. HRP-conjugated goat anti-mouse IgG was used as secondary antibody at a dilution of 1:5000.

### Citrus transformation

Epicotyl segments of Wanjincheng seedlings were used for *A. tumefaciens*-mediated transformation. The details of performance were as previously described (Peng et al., 2015). Transgenic shoots were determined by GUS staining before being micrografted onto Wanjingcheng seedlings *in vitro*. The resulting plantlets were further grafted onto *C. limon* in a greenhouse. The expression levels of *C*Las4425 in transgenic plants were validated with qRT-PCR.

### Pathogen inoculation

To analyze the effect of *C*Las4425 on *N. benthamiana* immunity, *N. benthamiana* plants were infiltrated with *C*Las4425, NLS : *C*Las4425, and GFP, respectively one day before DC3000 Δ*hopQ1-1* inoculation. DC3000 was prepared with OD_600 =_ 0.0001 (10^4^ cfu/mL) and injected into six *N. benthamiana* plants for each treatment. Leaves were detached at 3 dpi and used for symptom observation and bacterial growth analysis.

To determine the role of *C*Las4425in HLB pathogenicity, five independent *C*Las4425-overexpressed lines were used *via* a grafting method. WT Wanjingcheng plants of the same age were used as controls. Branches from each of these plants were inoculated by stems of *C*Las-infected sugar orange plants, with the presence of *C*Las confirmed by PCR. Leaf samples were collected monthly, and midribs from three mature leaves per plant were used for DNA extraction and the determination of *C*Las populations.

### H_2_O_2_ measurement

Quantification of H_2_O_2_ production was performed using Hydrogen Peroxide (H_2_O_2_) Content Assay Kit (Molfarming, Nanjing, China), and the OD was measured using Varioskan Flash Microplate Reader (Thermo Scientific, China). Fresh tissues were collected from fully mature leaves and finely ground with liquid nitrogen, and H_2_O_2_ accumulation was assayed following the manufacturer’s instructions. The experiment comprised three biological replicates and was repeated three times.

## Results

### *C*Las4425 is a Sec-dependent effector and expressed mainly *in planta*

**Figure 1:**
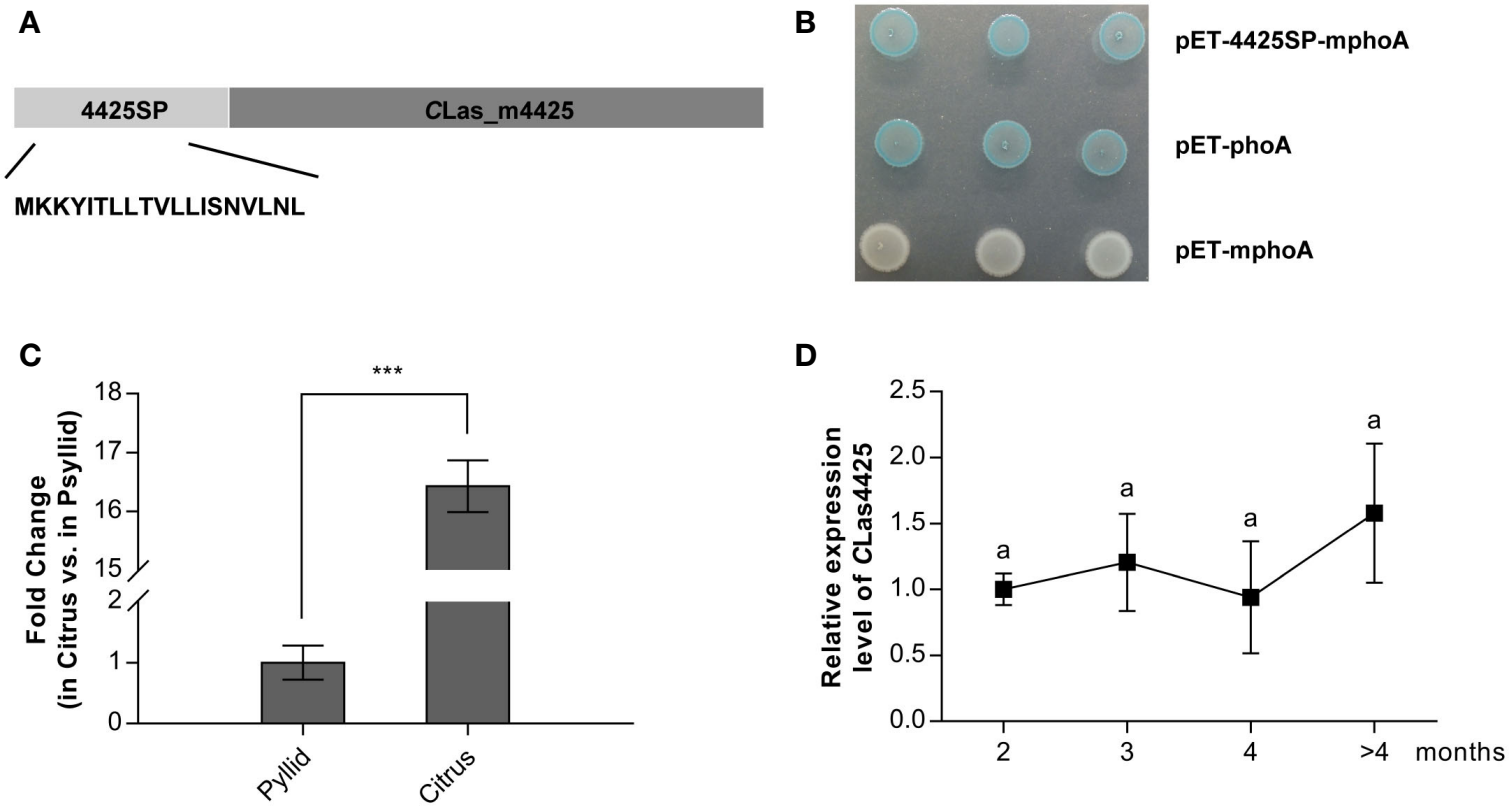
The secretion and expression analysis of *C*Las442*5*. **(A)**, Schematic of the *C*Las4425 protein. *In silico* analysis revealed a signal peptide at the N-terminus of *C*Las4425, 19 amino acids in length. 4425SP, the signal peptide of *C*Las4425; *C*Las_m4425, the mature form of *C*Las4425. **(B)** Alkaline phosphatase fusion assay confirmed the secretion of *C*Las4425. The *Escherichia coli* cells harboring the recombinant plasmids were incubated on Luria-Bertani (LB) plates amended with 5-bromo-4-chloro-3-indolyphosphate (BCIP) and sodium phosphate. As the negative control, pET-mphoA did not cause color change, while pET-phoA (positive control) and the recombinant turned to blue. **(C)**, Transcript levels of *CLas4425* in psyllid and citrus. Transcript levels analyzed with qRT-PCR were normalized to levels in psyllid using *CLasgyrA* (GenBank no. CP001677.5) as the internal reference. Means and standard errors from three biological replicates are shown. Asterisks reveal significant differences based on Student’s *t*-test (***p<0.001, n=3). **(D)**, Monitoring *C*Las4425 transcription in *C*Las-graft inoculated Wanjincheng (*C*. *sinesis*), measured using qRT-PCR and normalized to the level of *CLas4425* at 2 months after *C*Las successful colonization using *CLasgyrA* as the internal reference. Means and standard errors from three biological replicates are shown. Letters exhibit the differences as measured using Fisher’s LSD test (*p*=0.05, n=3).

An *in silico* analysis suggested *C*Las4425 contained a 19 amino signal peptide in the N-terminal (**Figure 1A**). Accordingly, an alkaline phosphatase (phoA) fusion assay was employed to validate the export of *C*Las4425 *via* the Sec translocon (Liu et al., 2019). After overnight incubation, *E*. *coli* cells harboring pET-4425SP-mphoA turned blue on indicator LB medium as the positive control, whereas the negative control remained white (**Figure 1B**). Based on these data, *C*Las4425 was identified as a typical Sec-delivered effector.

To explore the importance of *C*Las4425 to *C*Las pathogenicity, we investigated its expression profiles in *C*L*as*-infected citrus and psyllids, as well as during the disease process. As a result, *C*Las4425 was highly expressed (ca. 16-fold) in infected citrus plants compared to that in psyllids (**Figure 1C**). In addition, *C*Las4425 could be detected at 2 months after *C*Las colonization, and its expression level stayed the same during disease development (**Figure 1D**). Our findings imply that *C*Las4425 plays a critical role in *planta* at early infection stage.

### *C*Las4425-triggered cell death in *N*. *benthamiana* is dependent on subcellular localization and the cytoplasmic receptor BIK1

**Figure 2:**
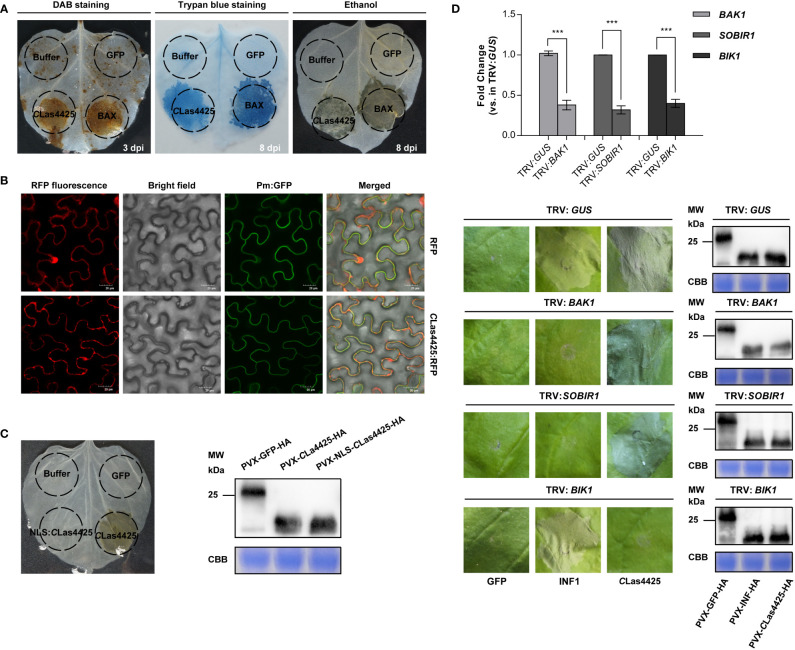
Analysis of impact factors in determination of *C*Las4425-triggered hypersensitive response (HR) in leaves of *Nicotiana benthamiana*. **(A)**, Programmed cell death and ROS burst induced by *C*Las4425. Leaves were infiltrated with inoculation buffer, PVX-GFP, PVX*-*BAX, and PVX-*C*Las4425. To visualize ROS burst, leaves were detached at 3 dpi for 3, 3’-diaminobenzidine (DAB) staining. The leaves for cell death observation were photographed at 8 days’ post inoculation (dpi) after trypan blue staining and ethanol decolorization. The experiment had three independent biological replicates and was repeated three times. **(B)**, Subcellular localization analysis. *C*Las4425 fused with RFP, the signals were detected at 3 dpi using a confocal laser scanning microscope. RFP was used as the location control while Pm : GFP was used as plasma membrane location marker. Scale bar: 20 μm. The experiment was repeated three times. **(C)**, Subcellular localization is required for *C*Las4425-triggered cell death. Corresponding proteins were expressed in *N*. *benthamiana* to assay triggering of cell death. Symptoms were photographed at 8 dpi after ethanol decolorization. Immunoblot of proteins from infiltrated leaves transiently expressing the indicated proteins from a PVX-3*HA vector. NLS, nuclear localization signal. **(D)**, *C*Las4425-induced cell death relies on a cytoplasmic receptor, BIK1. *N*. *benthamiana* plants were subjected to virus-induced gene silencing (VIGS) by inoculation with TRV : *GUS*, TRV : *BAK1*, TRV : *SOBIR1*, and TRV : *BIK1*, respectively. The expression levels of silenced genes after VIGS treatment were determined by qRT-PCR analysis using the *NbACTIN* endogenous control (Student’s *t*-test, ***p<0.001, n=6). Three weeks after inoculation, GFP, INF1, and *C*Las4425 were transiently expressed in the gene-silenced *N*. *benthamiana*, and then leaves were photographed at 8 dpi. Immunoplot analysis of GFP, INF1, and *C*Las4425 protein fused with 3*HA tags transiently expressed in VIGS-silenced *N*. *benthamiana* plants. The experiment was performed three times with six plants for each TRV construct.

A transient expression system was applied to assess the potential role of *C*Las4425 in *C*Las virulence. The coding sequence of *C*Las4425, without the secretion signal, was cloned into the binary PVX vector pGR107. PVX-GFP and PVX-BAX served as negative and positive control, respectively. Compared to BAX, *C*Las4425 induced mild cell death symptom (**Figure 2A**). The progression, in which *C*Las4425-triggered cell death happened, was recorded (**Figures S1A, B**). To avoid the influence of PVX, we sequentially constructed pLGN-*C*Las4425. Cell death was also observed in *C*Las4425-infiltrated leaves (**Figure S1C**).

Protein functions are correlated with their subcellular localization. To visualize the distribution of *C*Las4425 in plant cells, we transiently expressed RFP and *C*Las4425:RFP in *N. benthamiana* leaves, and observed their localization pattern with a confocal microscope. Free RFP was found to be distributed in multiple subcellular compartments (**Figures 2B**; **S2**), while *C*Las4425:RFP was localized in the cytoplasm and cytoplasmic membrane (**Figure 2B**). To reveal the role of subcellular localization involved in *C*Las4425-triggered cell death, the nuclear localization sequence (NLS) (Liu et al., 2019) was fused in frame to *C*Las4425 in PVX (**Figure S2**). Transient expression illustrated that NLS : *C*Las4425 could not induce cell death, and the protein expression was validated (**Figure 2C**). Therefore, localization in cytoplasm and cytoplasmic membrane is required for *C*Las4425-activated cell death.

Brassinosteroid insensitive 1-associated kinase 1 (BAK1), a suppressor of BIR1-1 (SOBIR1), and BIK1 participate in several types of PTI signaling pathways (Liebrand et al., 2014; Kadota et al., 2015). Moreover, BIK1 is necessary for bacterial resistance during ETI (Yuan et al., 2021). As a PAMP, INF1-induced cell death relies on *BAK1* and *SOBIR1* (Heese et al., 2007; Franco-Orozco et al., 2017). Here, we expressed *C*Las4425 in *N. benthamiana* silenced for *BAK1*, *SOBIR1*, and *BIK1.* The silencing of these genes was verified using qRT-PCR (*p*<0.001) (**Figure 2D**). *C*Las4425-triggered cell death developed in the TRV : *BAK1*, TRV : *SOBIR1*, and TRV : *GUS* treated plants. On the contrary, cell death was abolished in *BIK1*-silenced *N. benthamiana* plants. However, INF1-activated cell death was not altered in TRV : *BIK1-*treated plants. Immunoblot verified that GFP, INF1, and *C*Las4425 were correctly expressed in *N. benthamiana*. Conclusively, *C*Las4425-elicited cell death is dependent on BIK1.

### *C*Las4425 hinders plant defense responses in *N*. *benthamiana*

**Figure 3:**
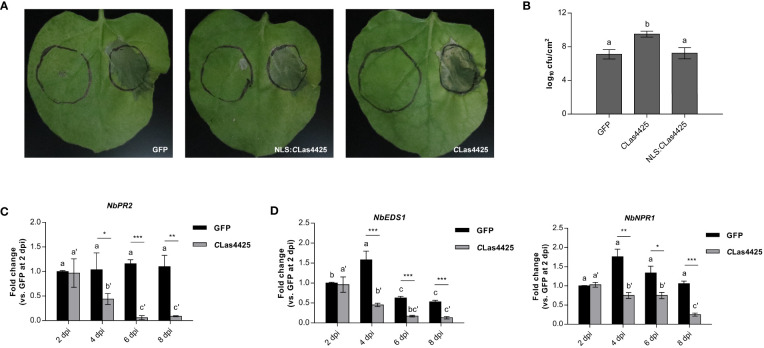
*C*Las4425 promotes the infection of *Pseudomonas syringae* pv. tomato (DC3000) Δ*hopQ1-1* in *N*. *benthamiana*. **(A)**, The symptoms of *N*. *benthamiana* leaves inoculated with DC3000 Δ*hopQ1-1*. Leaves were infiltrated with GFP, *C*Las4425, and NLS: *C*Las4425 one day before inoculation with DC3000 Δ*hopQ1-1*. The photographs were taken at 3 dpi. The experiment was repeated three times, and at each time six independent biological replicates were taken for agro-infiltration with corresponding plasmids. **(B)**, Colony-forming units per square centimeter (cfu/cm^2^) were counted to assess the bacterial population. Significant difference was determined with Fisher’s LSD test (*p*=0.05, n=3). **(C)**, qRT-PCR analysis of *NbPR2* after PVX-*C*Las4425 infiltration compared with PVX-GFP. **(D)**, The expression of SA upstreaming genes *NbEDS1* and *NbNPR1*. In **(C, D)** the leaves were collected for RNA extraction of *C*Las4425 or GFP control at 2, 4, 6, and 8 dpi. The transcript levels measured with qRT-PCR were normalized to levels in GFP at 2 dpi using the *NbACTIN* endogenous control. The differences between *C*Las4425- and GFP-expressing samples were analyzed using Student’s *t*-test (*p<0.05, ** *p*<0.01, ***p<0.001, n=3). Fisher’s LSD test was used to compare gene expression within the timelines (*p*=0.05, n=3).

DC3000 Δ*hopQ1-1* was used to determine the virulence of *C*Las4425. We inoculated *N*. *benthamiana* leaves with DC3000 following the infiltration with either *C*Las4425 or NLS : *C*Las4425. qRT-PCR analysis revealed that the PVX accumulation level of PVX-*C*Las4425 made no significant difference to PVX-GFP in *N*. *benthamiana* (**Figure S3A**). *N*. *benthamiana* expressing *C*Las4425 exhibited more severe leaf blight than those infiltrated with NLS : *C*Las4425 and GFP (**Figure 3A**). Analysis of bacterial growth revealed that *C*Las4425 promoted DC3000 proliferation in *N*. *benthamiana* (**Figure 3B**). Since *C*Las4425 had a negative effect on *N*. *benthamiana* immunity reacting to DC3000 infection, we probed the molecular evidence for its interference with plant innate immunity. The expression of a PTI marker gene, *NbWRKY29*, and genes representing phytohormones were detected (Pang et al., 2020; Situ et al., 2020). The transcript of *NbPR2* declined significantly from 4 dpi (**Figure 3C**), whereas the expression level of other detected genes did not vary dramatically (**Figure S3B**). Considering *NbPR2* marked the SA pathway, we examined the expression manners of SA upstreaming genes, namely *NbEDS1* and *NbNPR1*. As expected, their expressions were decreased in plants expressing *C*Las4425 (**Figure 3D**). Hence, *C*Las4425 probably impairs *N*. *benthamiana* defense *via* targeting the SA signaling pathway.

### *C*Las4425 enhances citrus susceptibility to *C*Las infection

**Figure 4:**
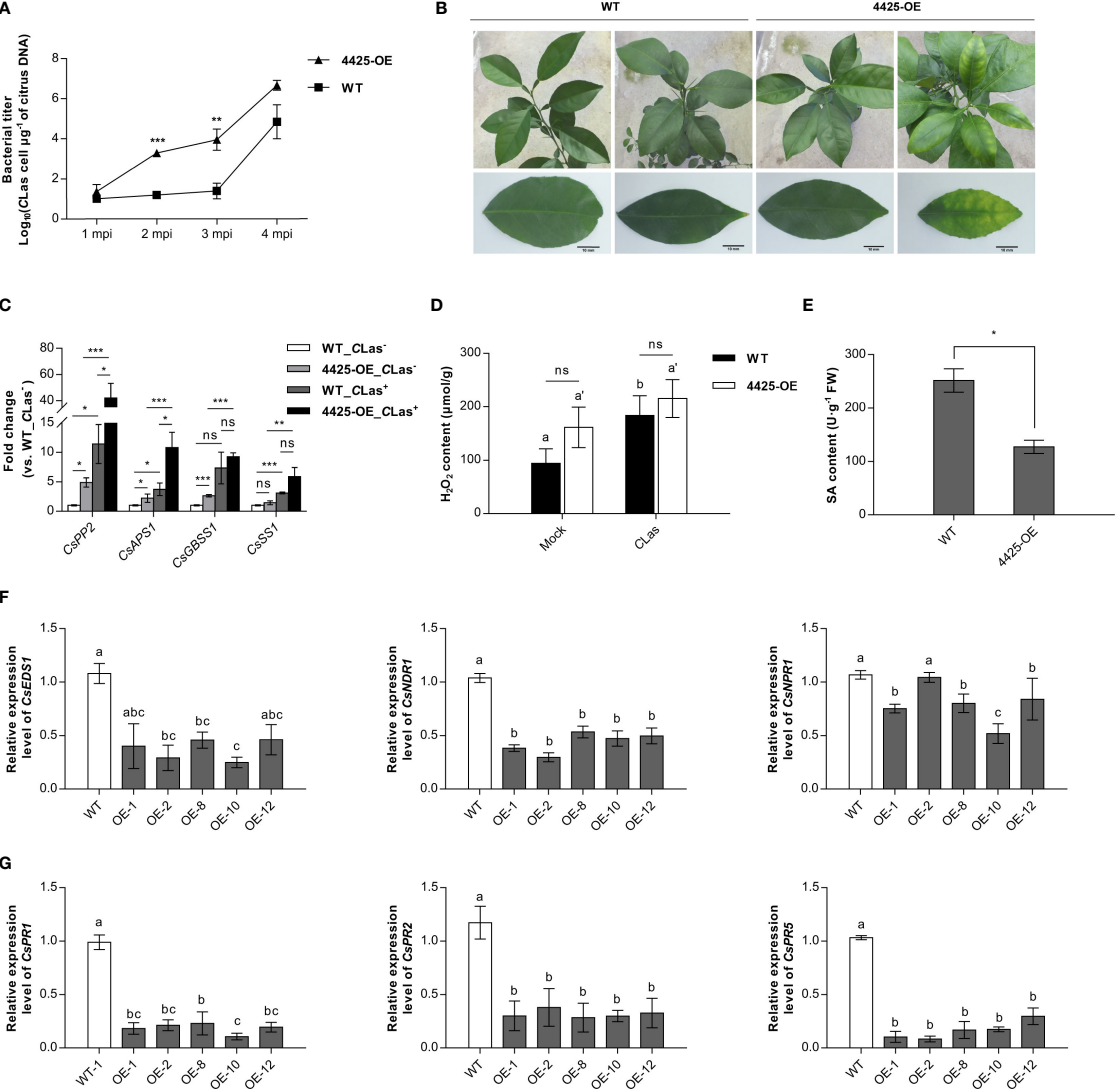
*C*Las4425 accelerates ‘*Candidatus* Liberibacter asiaticus’ (*C*Las) colonization and symptom development in the transgenic citrus plants. **(A)**, Quantitative analysis of *C*Las growth during a 4-month period after graft inoculation in *C*Las4425 transgenic citrus lines (4425-OE) and wild-type plants (WT). Wanjincheng (*C*. *sinesis*) were used for generation of *C*Las4425 transgenic citrus. Wild-type Wanjincheng of the same age were used as controls. MPI=months post-inoculation. Transgenic and WT plants contained five independent biological replicates each. The bacterial populations (*C*Las cells μg^-1^ of citrus DNA) were determined using qPCR. Asterisks represent significant differences based on Student’s *t*-test (**p<0.01, ***p<0.001, n=5). **(B)**, Leaves of 4425-OE lines exhibited a blotchy mottle at 6 MPI. Scale bar: 10 mm. **(C)**, Transcript levels of genes associated with phloem blockage (*CsPP2*), sugar metabolism (*CsAPS1*), and starch granule accumulation (*CsGBSS1* and *CsSS1*) in *C*Las-infected plants. Transcript levels measured with qRT-PCR were normalized to levels in *C*Las-infected WTs using the *CsGAPDH* endogenous control. The differences were analyzed using Student’s *t*-test (***p<0.001, n=3). **(D)**, H_2_O_2_ accumulation in transgenic and WT plants at 6 MPI. Significant differences are based on Student’s *t* test (n=3). The asterisks indicate the differences between the transgenic and WT plants (ns, no significance), and the letters indicate the significant differences between the mock and *C*Las-infected samples (*p*<0.05). **(E)**, SA accumulation in transgenic and WT plants at 6 MPI. The differences between the transgenic and WT plants were analyzed using Student’s *t*-test (*p<0.05, n=3). **(F)**, Transcript levels of SA-mediated signal transduction-related genes. **(G)**, Transcript levels of pathogenic-related (*PR*) genes. In **(F, G)**, transcript levels measured with qRT-PCR were normalized to levels in WT plants using the *CsGAPDH* endogenous control. Letters exhibit the differences as measured using Fisher’s LSD test (*p*=0.05, n=3).

To assess the significance of *C*Las4425 in *C*Las pathogenicity, we generated *C*Las4425*-*transgenic Wanjincheng (4425-OE). The obtained transgenic plants were clarified using PCR, qRT-PCR, and GUS assay (**Figure S4A**), and there was no difference between 4425-OEs and wild-type group (WT) in growth and phenotype (**Figure S4B**). Among seven independent transgenic lines, we selected five of them with relatively higher expression of *C*Las4425 for further studies. The five 4425-OEs (one-year old) were graft-inoculated using *C*Las-infected stems. Five WTs of the same age served as a control group and were grafted in the same way. qPCR was applied monthly to monitor bacterial growth. Comparison of bacterial titers demonstrated that *C*Las growth was faster in 4425-OEs than in WTs at 2 (*p*<0.001) and 3 months post inoculation (MPI) (*p*<0.01), whereas no difference was observed after 4 MPI (*p*>0.05) (**Figure 4A**). Leaves from transgenic plants exhibited a blotchy mottle at 6 MPI, while WTs remained asymptomatic (**Figure 4B**). Previous reports documented the accumulation of starch granules and phloem blockage in infected citrus plants (Wang and Trivedi, 2013). We therefore detected the transcription of genes associated with phloem protein (*CsPP2*), sugar metabolism (*CsAPS1*), and starch biosynthesis (*CsGBSS1* and *CsSS1*) (Kim et al., 2009). Consequently, *C*Las promoted the expression of all the detected genes in both WTs and 4425-OEs while *C*Las4425 enhanced the expression of *CsPP2*, *CsAPS1*, and *CsGBSS1* in uninfected citrus plants. Compared to those in WTs infected with *C*Las, the expression of *CsPP2* and *CsAPS1* was upregulated in *C*Las-infected 4425-OEs (**Figure 4C**). To sum up, *C*Las4425 promotes *C*Las multiplication, and enhances HLB progression.

To understand how *C*Las4425 promotes *C*Las colonization, the H_2_O_2_ and SA levels in citrus were further investigated. The H_2_O_2_ content was not significantly different between 4425-OEs and WTs. Of interest, *C*Las stimulated H_2_O_2_ accumulation in WTs but not in 4425-OEs (**Figure 4D**). Herein, *C*Las4425 stimulated a slight increase of H_2_O_2_ in citrus plants, which is consistent with mild HR induced by *C*Las4425 in *N. benthamiana*. Moreover, *C*Las4425 hindered SA accumulation (*p*<0.05) (**Figure 4E**). We then detected the expression of SA signaling pathway genes. The expression of *CsEDS1*, *CsNDR1*, *CsNPR1*, *CsPR1*, *CsPR2*, and *CsPR5* was depressed in 4425-OE lines (**Figures 4F, G**). Therefore, *C*Las4425 suppresses the plant immune system through interfering with the SA signaling pathway.

## Discussion

Since the release of the first *C*Las genome in 2009, the effector and host interactions have become a hotspot for studying *C*Las pathogenesis. Vectored by ACP, *C*Las utilizes the effector repertoire to adjust to distinct insects and plants, in addition to different disease stages. Analysis of global gene expression changes in *C*Las during transmission between citrus and psyllid suggests that the genes up-regulated *in planta* are mainly involved in the virulence and/or survival of the pathogen (Yan et al., 2013). Consistent with this suggestion, the expressions of effectors, including *C*Las4425 and previously reported virulence factors, remarkably increased *in planta* compared to those in psyllids (Liu et al., 2019; Du et al., 2022; Shen et al., 2022). Additionally, temporal detection of effector transcripts is an important aspect of the study on pathogen effectors during the initial microbe-plant interactions (Shi et al., 2019). In this study, the expression level of *CLas4425* was detected after two months of *C*Las inoculation and maintained the same level during the infection processes. The consistent expression of *C*Las4425 could act as a virulence factor to create a favorable phloem environment for *C*Las proliferation and enhancement of HLB symptom.

Transient expression of *C*Las4425 elicited cell death in *N*. *benthamiana*. Instead of inducing obvious symptoms in citrus plants, *C*Las4425 stimulated the expression of *CsPP2*, which plays a critical role in plant defense responses to biotic stresses (Zhang et al., 2011; Will et al., 2013). Previous reports demonstrated that SDE1 and AGH17470 are also HR elicitors, while most *C*Las effectors were identified to suppress defense responses (Pitino et al., 2016; Zhang et al., 2020; Du et al., 2022; Oh et al., 2022). LasBCP, SC2_gp095, and SECP8 were reported as ROS scavengers, and AGH17488 targets APX6 to eliminate ROS (Jain et al., 2015; Jain et al., 2019; Shen et al., 2022; Du et al., 2023). The phenomenon indicated effector-effector interactions, which may be due to a co-pathway targeted by the ETI-eliciting and ETI-suppressing effectors (Zhang et al., 2015; Martel et al., 2018). The interactions could result in the long incubation period before visible HLB symptoms. However, the interaction mechanism remains to be elucidated.

Effector-triggered cell death is tightly related to subcellular localization (Yu et al., 2012; Asai et al., 2018). *C*Las4425 exclusively localized to cytoplasm and cytoplasmic membrane, and the enforcement of its nuclear accumulation impaired its ability to initiate cell death. Intriguingly, silencing of the cytoplasmic receptor NbBIK1 rather than the membrane receptors NbBAK1 and NbSOBIR1 also perturbed *C*Las4425 cell death-inducing activity. In *Arabidopsis*, BIK1 is required for phosphorylation of respiratory burst oxidative homolog D (RBOHD) in PTI and ETI-mediated ROS generation (Yuan et al., 2021). Hence, *C*Las4425 is speculated to indirectly activate BIK1 to phosphorylate RBOHB, leading to ROS burst and then cell death *via* ETI regulation.

ETI leads to a complex array of global as well as specific defense responses, which require the participation of phytohormones (Zhang and Li, 2019). Among these, SA serves as a key plant defense hormone, and is required for the establishment of systemic acquired resistance (SAR) (Dempsey et al., 2011; Tanaka et al., 2015). The SA-associated pathway is sophisticatedly manipulated. For instance, EDS1 and NDP1 have been identified as upstream regulators of SA biosynthesis during ETI (McDowell et al., 2000; Chang et al., 2022). Increasing levels of SA accelerate NPR1 movement to the nucleus, which results in activating the expression of SA-dependent pathogenesis-related genes, *PR1*, *PR2*, and *PR5* (Ali et al., 2018; Chen et al., 2021). Evidence showed that SA accumulation increases in the late stage of *C*Las infection, and artificially increasing SA levels can elevate HLB-tolerance (Li et al., 2021; Peng et al., 2021). Correspondingly, *C*Las have evolved efficient tactics to downregulate the SA-signaling pathway. For instance, *C*Las-encoded SahA serves as an SA hydroxylase, and SDE1 targeted citrus PLCPs proteases that were related to SA-induced defense (Li et al., 2017; Clark et al., 2018). The repressed SA level and related genes expression in both *N. benthamiana* and citrus plants revealed that *C*Las4425 may confer broad-spectrum virulence through regulating the SA-signaling pathway. Nevertheless, the insight into the role of *C*Las4425 in pathogenicity remains to be unveiled.

## Conclusion

*C*Las pathogenesis is of great importance but challenging to study because of its phloem-limited nature, intracellular lifestyle, and inability to be cultured. In the study, *C*Las4425 was identified to be expressed at the early stage of *C*Las infection, and could play a critical role in *C*Las multiplication. *C*Las4425 expression results in HR in *N*. *benthamiana*, which relies on its subcellular localization and the presence of BIK1. However, *C*Las4425 interferes with the SA-mediated signaling pathway and promotes *C*Las infection and disease development.

## Data availability statement

The original contributions presented in the study are included in the article/**Supplementary Material**. Further inquiries can be directed to the corresponding authors.

## Author contributions

SSZ, TZ, Shimin Fu, and CZ designed the experiments. SSZ performed the experiments, analyzed the data, as well as wrote the draft manuscript. JH, XW, SZ, SF, and CZ revised and polished the manuscript. All authors contributed to the article and approved the submitted version.

## Supplementary material

Supplementary Table 1 Primer pairs used in this study.

Supplementary Figure 1 The progression of *C*Las4425-triggered cell death. **(A)**, The records of *C*Las4425-triggered cell death at 3-8 dpi with cell staining and ethanol decolorization. The diagram on the left side represents the sites infiltrated with inoculation buffer, PVX-GFP, PVX*-*BAX, and PVX-*C*Las4425, respectively. The experiment had three independent biological replicates and was repeated twice with similar results. **(B)**, H_2_O_2_ contents in *N*. *benthamiana* expressing GFP and *C*Las4425. Leaves were collected at the indicated time points. The differences between *C*Las4425- and GFP-expressing samples were analyzed using Student’s *t*-test (**p*<0.05, ** *p*<0.01, n=3). Dunnett’s T3 test was used to compare H_2_O_2_ accumulation within the timelines (*p*=0.05, n=3). **(C)**, *C*Las4425-inducing cell death activity clarified using pLGN vector. Leaves were infiltrated with *Agrobacterium tumefaciens* carrying pLGN-GFP, pLGN-*C*Las4425, and PVX-BAX, and then photographed at 8 dpi.

Supplementary Figure 2 NLS : *C*Las4425:RFP verified using the confocal laser scanning microscope. RFP was used as the location control while H2B:GFP was used as the nucleus marker. Scale bar: 20 μm.

Supplementary Figure 3 Transcript levels of PVX and genes associated with plant innate immunity. **(A)**, Accumulation of PVX in *N*. *benthamiana*. **(B)**, Transcript levels of a PAMP-triggered immunity gene (*NbWRKY29*) and the genes corresponding to jasmonic acid and ethylene signaling pathways, namely *NbLOX* and, *NbERF1*. In **(A, B)**, qRT-PCR was used to analyze gene expression in response to *C*Las4425 infiltration. The transcript levels were normalized to levels in GFP at 2 dpi using the *NbACTIN* endogenous control. The differences between *C*Las4425- and GFP-expressing samples were analyzed using Student’s *t*-test (**p*<0.05, ***p*<0.01, ****p*<0.001, n=3). Fisher’s LSD test was used to compare gene expression within the timelines (*p*=0.05, n=3).

Supplementary Figure 4
**(A)**, Construction of *C*Las4425-transgenic citrus plants. Structure of the pLGN-*CLas4425* plasmid used for the overexpression assays. Identification of transgenic plants with PCR, RT-qPCR, and β-glucuronidase (GUS) histochemical staining. M, DNA marker; OE, transgenic citrus plants; WT, wildtype control. Scale bar: 9 mm. **(B)**, Phenotypes of WT and 4425-OE. Scale bar: 10 cm.
