# Supplementary material for: A Sec-dependent effector, CLIBASIA_04425, contributes to virulence in ‘Candidatus Liberibater asiaticus’
Source: Front Plant Sci. 2023 Jul 24;14:1224736. doi: 10.3389/fpls.2023.1224736 (PMC10405523; doi:10.3389/fpls.2023.1224736)
Supplement: Supplementary Table 1 — Primer pairs used in this study. [file Table_1.docx]

| **Purpose** | **Plasmid** | **Primer sequence (5'-3')** |  | **Restrictrion enzyme** |
| --- | --- | --- | --- | --- |
| Transient expression in *N. benthamiana* | PVX-HA | F | accagctagcatcgatATGTACCCATACGAT | *Cla*I |
|  |  | R | ttcatcggcggtcgaccttaAGCGTAATCTGG | *Sal*I |
|  | PVX-GFP-HA | F | tcagcaccagctagcatcgatATGGTGAGCAAGGGCGAGG | *Cla*I |
|  |  | R | atcgtatgggtacatatcgatAAGATCTACCATGTACAGCTCGTCC |  |
|  | PVX-*C*Las4425-HA | F | tcagcaccagctagcatcgatATGTATGATGCGAAAGCAAGAAGA |  |
|  |  | R | atcgtatgggtacatatcgatTGGGATCGGTAGTTTCGATAATATC |  |
|  | PVX-NLS-*C*Las4425-HA | F | tcagcaccagctagcatcgat ATGCCTAAGAAGAAGAGAAAGGTTTATGATGCGAAAGCAA |  |
|  |  | R | atcgtatgggtacatatcgatTGGGATCGGTAGTTTCGATAATATC |  |
|  | PVX-INF1-HA | F | tcagcaccagctagcatcgatATGAACTTTCGTGCTCTGTTCGC |  |
|  |  | R | atcgtatgggtacatatcgatTAGCGACGCACACGTAGACG |  |
|  | PVX-RFP | F | accagctagcatcgatATGGCCTTCTCCGAGGACG | *Cla*I |
|  |  | R | gcatttctggaagcttTTAGGCGCCGGTGGAGTGGCG | *Sal*I |
|  | PVX-*C*Las4425-RFP | F | accagctagcatcgatATGTATGATGCGAAAGCAAGAAGA | *Cla*I |
|  |  | R | ctcggagaaggccatatcgatCTGGGATCGGTAGTTTCGATAATAT |  |
|  | PVX-NLS-*C*Las4425-RFP | F | tcagcaccagctagcatcgatATGCCTAAGAAGAAGAGAAAGGTTTAT |  |
|  |  | R | ctcggagaaggccatatcgatCTGGGATCGGTAGTTTCGATAATAT |  |
|  | pLGN-GFP | F | ggacagggtacccggggatcc atggtgagcaagggcgaggag | *Bam*HI |
|  |  | R | agggaattcctgcaggtcgac tcaaagatctaccatgtacag | *Sal*I |
|  | pLGN-BAX | F | ggacagggtacccggggatcc ATGGACGGGTCCGGG | *Bam*HI |
|  |  | R | agggaattcctgcaggtcgac TCAAGCGTAATCTGG | *Sal*I |
| *Citrus sinesis* transformation | pLGN-*C*Las4425 | F | atccactagtgtcgacatgTATGATGCGAAAGCAAGAAGATTCC | *Bam*HI |
|  |  | R | ttaaagcagggaattcTTACTGGGATCGGTAGTTTCGATAATATCCAT | *Sal*I |
| Secretory clarification | *C*Las4425_sp-mphoA | F | aaggagatatacatatgATGAAGAAGTATATCACATTATTAACAGTATTACTCATAAGTAACGTGCTGAACCTG | *Nde*I |
|  |  | R | gcatttctggaagcttCAGGTTCAGCACGTTACTTATGAGTAATACTGTTAATAATGTGATATACTTCTTCAT | *Hin*dIII |
| VIGS assay in *N. benthamiana* | TRV*:BAK1* | F | GTGAGTAAGGTTACCGAATTCGTGAGGGTGGTGAGCAGGATA | *Eco*RI |
|  |  | R | GGGACATGCCCGGGCCTCGAG AGCTCATAACTGGGCAACGG | *Xho*I |
|  | TRV*:SOBIR1* | F | GTGAGTAAGGTTACCGAATTC CAGAAAGTTTTCCAATGGCAG | *Eco*RI |
|  |  | R | GGGACATGCCCGGGCCTCGAG AATCTTTATCCACCAGATCAT | *Xho*I |
|  | TRV*:BIK1* | F | GTGAGTAAGGTTACCGAATTC TCATCTTCTAAGCAATATCCA | *Eco*RI |
|  |  | R | GGGACATGCCCGGGCCTCGAG AGCTGAGTCTCCTCTTCATGC | *Xho*I |
|  | TRV*:GUS* | F | GTGAGTAAGGTTACCGAATTCcgtactcggatggaa | *Eco*RI |
|  |  | R | GGGACATGCCCGGGCCTCGAGcgtcaagaaggcgat | *Xho*I |
| *C*Las detection | *16S rDNA-OI1* | F | GCGCGTATGCAATACGAGCGGCA |  |
|  | *16S rDNA-O2c* | R | GCCTCGCGACTTCGCAACCCAT |  |
| Quantitative PCR | *CLasgyrA* | F | GTATGGCACAGGACTGGTCT |  |
|  |  | R | GTTAGGGCGGAAATCAACAGT |  |
|  | *18S rRNA* | F | AATTGTTGGTCTTCAACGAGGAA |  |
|  |  | R | AAAGGGCAGGGACGTAGTCAA |  |
|  | *q4425* | F | CTCATAAGTAACGTGCTGAACCTGT |  |
|  |  | R | GTTGCTATACGTTCTTCAGATCCAT |  |
|  | *CsGAPDH* | F | CATCCCTCAGCACCTTCC |  |
|  |  | R | CCAACCTTAGCACTTCTCC |  |
|  | *qNbBAK1* | F | ATTGCTGGAGGAGTTGCTGCCGG |  |
|  |  | R | CCACGTACAGCAGTGGTAACAT |  |
|  | *qNbSOBIR1* | F | CTTAGAAAAACTCTCTTTAGC |  |
|  |  | R | TATGGATTGGAGTGACATTATG |  |
|  | *qNbBIK1* | F | CTCCGGATGAAGGTTGCTCT |  |
|  |  | R | CTCGGGAGCAGCATAACCAT |  |
|  | *qNbACTIN* | F | CAGCTCATCCGTGGAGAAGA |  |
|  |  | R | AGGATACGGGGAGCTAATGC |  |
|  | *qNbPR2* | F | GCACGACATAACCTTCCACTCTTAG |  |
|  |  | R | ACCCTGCTGAATTTGTTCCTTG |  |
|  | *qNbEDS1* | F | TGGAAATGGGAAACTGGTGGTC |  |
|  |  | R | GACAAGGGAATATCGGTAAGATTATTG |  |
|  | *qNbNPR1* | F | GAAACGCCTATCGGAAACACTG |  |
|  |  | R | AAGCCAATACACTCATTACAGCATC |  |
|  | *qCsPR1* | F | AAATGTGGGTGAATGAGAAAGC |  |
|  |  | R | ATTATTGTTGCACGTCACCTTG |  |
|  | *qCsPR2* | F | TTCCACTGCCATCGAAACTG |  |
|  |  | R | GTAATCTTGTTTAAATGAGCCTCTTG |  |
|  | *qCsPR5* | F | CACCATTGCCAATAACCCTAATG |  |
|  |  | R | GGGACAGTTACCGTTAAGATCAG |  |
|  | *qCsNPR1* | F | AGAGGACCCAAGTTTGAGCT |  |
|  |  | R | CAGGCATCATCATCCACAC |  |
|  | *qCsEDS1* | F | CACCAGCAGAATGCCACCTA |  |
|  |  | R | GCATTGCTCATCACCGTCAC |  |
|  | *qCsNDR1* | F | TCATGTGGCTGAGTCTACGC |  |
|  |  | R | GATGTGGCTTCGATTGGGGA |  |
