# Supplementary figures and images for: A Sec-dependent effector, CLIBASIA_04425, contributes to virulence in ‘Candidatus Liberibater asiaticus’
Source: Front Plant Sci. 2023 Jul 24;14:1224736. doi: 10.3389/fpls.2023.1224736 (PMC10405523; doi:10.3389/fpls.2023.1224736)

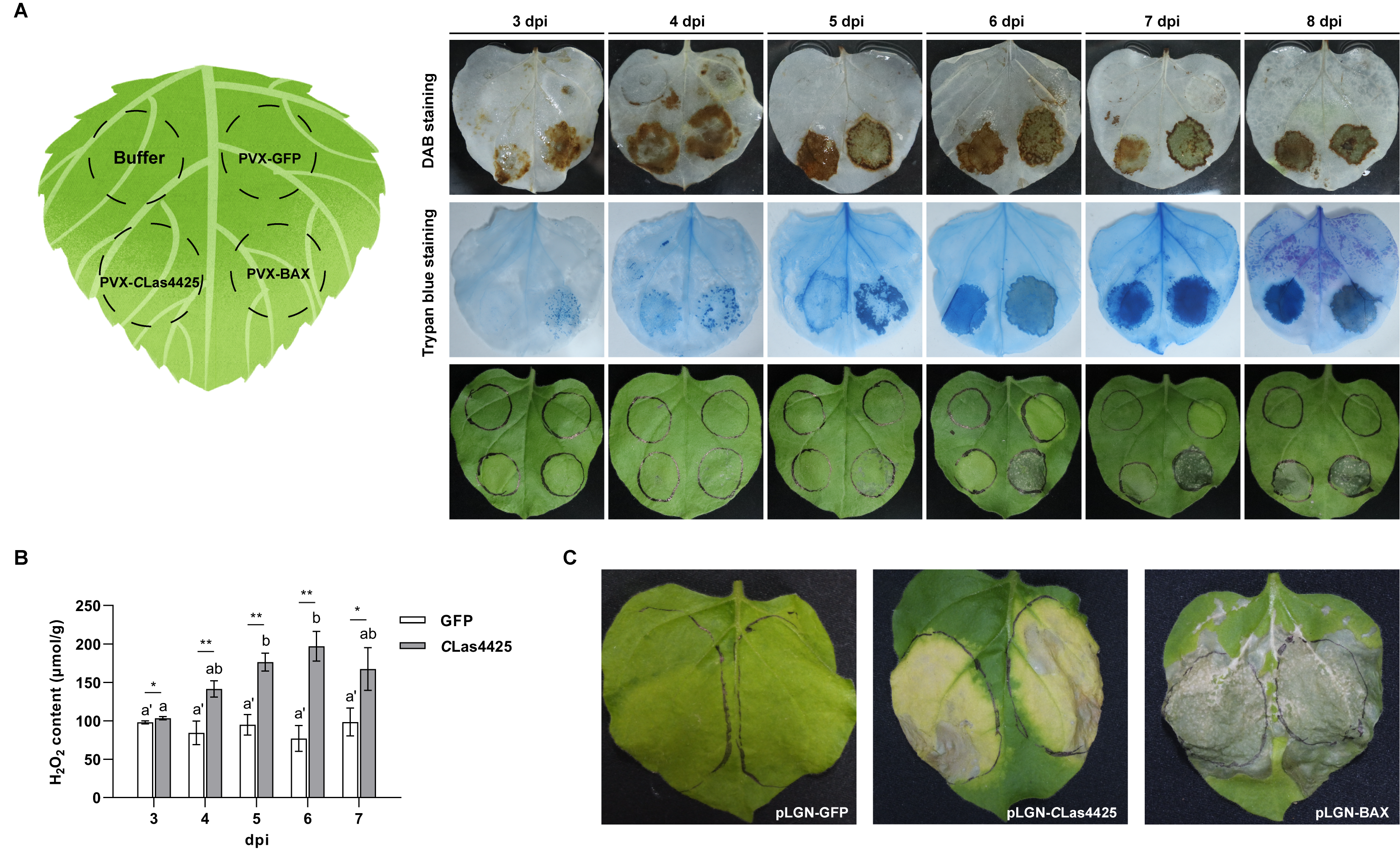

Supplement: Supplementary Figure 1 — The progression of CLas4425-triggered cell death. (A), The records of CLas4425-triggered cell death at 3-8 dpi with cell staining and ethanol decolorization. The diagram on the left side represents the sites infiltrated with inoculation buffer, PVX-GFP, PVX-BAX, and PVX-CLas4425, respectively. The experiment had three independent biological replicates and was repeated twice with similar results. (B), H2O2 contents in N. benthamiana expressing GFP and CLas4425. Leaves were collected at the indicated time points. The differences between CLas4425- and GFP-expressing samples were analyzed using Student’s t-test (*p<0.05, ** p<0.01, n=3). Dunnett’s T3 test was used to compare H2O2 accumulation within the timelines (p=0.05, n=3). (C), CLas4425-inducing cell death activity clarified using pLGN vector. Leaves were infiltrated with Agrobacterium tumefaciens carrying pLGN-GFP, pLGN-CLas4425, and PVX-BAX, and then photographed at 8 dpi. [file Image_1.tif]

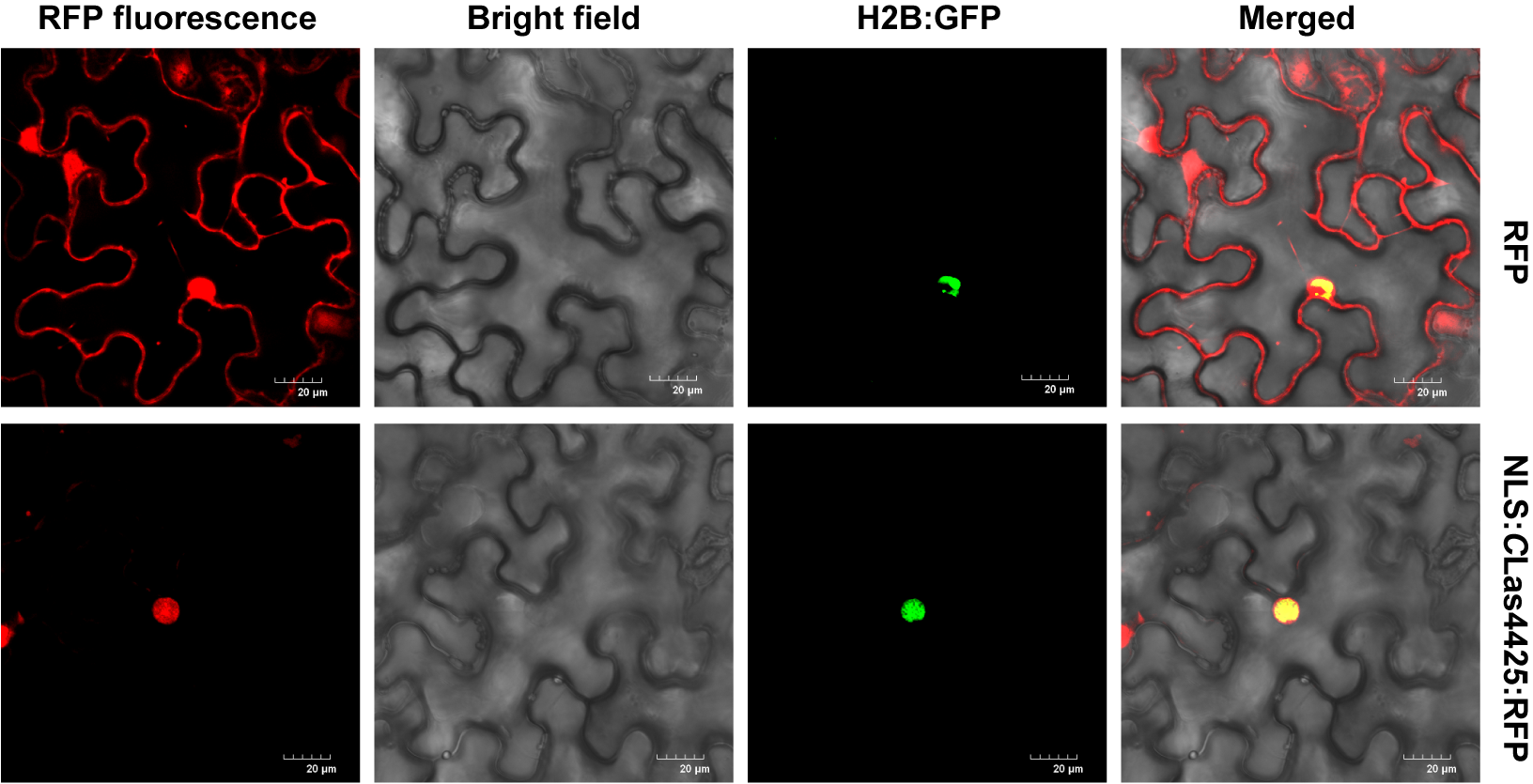

Supplement: Supplementary Figure 2 — NLS : CLas4425:RFP verified using the confocal laser scanning microscope. RFP was used as the location control while H2B:GFP was used as the nucleus marker. Scale bar: 20 μm. [file Image_2.tif]

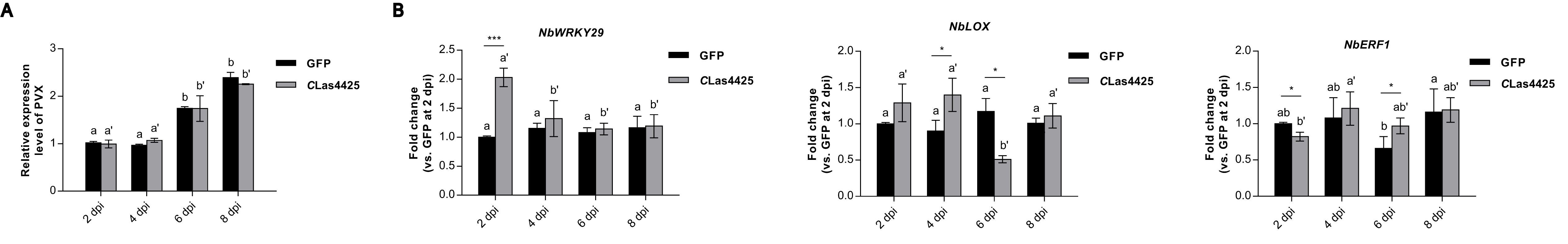

Supplement: Supplementary Figure 3 — Transcript levels of PVX and genes associated with plant innate immunity. (A), Accumulation of PVX in N. benthamiana. (B), Transcript levels of a PAMP-triggered immunity gene (NbWRKY29) and the genes corresponding to jasmonic acid and ethylene signaling pathways, namely NbLOX and, NbERF1. In (A, B), qRT-PCR was used to analyze gene expression in response to CLas4425 infiltration. The transcript levels were normalized to levels in GFP at 2 dpi using the NbACTIN endogenous control. The differences between CLas4425- and GFP-expressing samples were analyzed using Student’s t-test (*p<0.05, **p<0.01, ***p<0.001, n=3). Fisher’s LSD test was used to compare gene expression within the timelines (p=0.05, n=3). [file Image_3.tif]

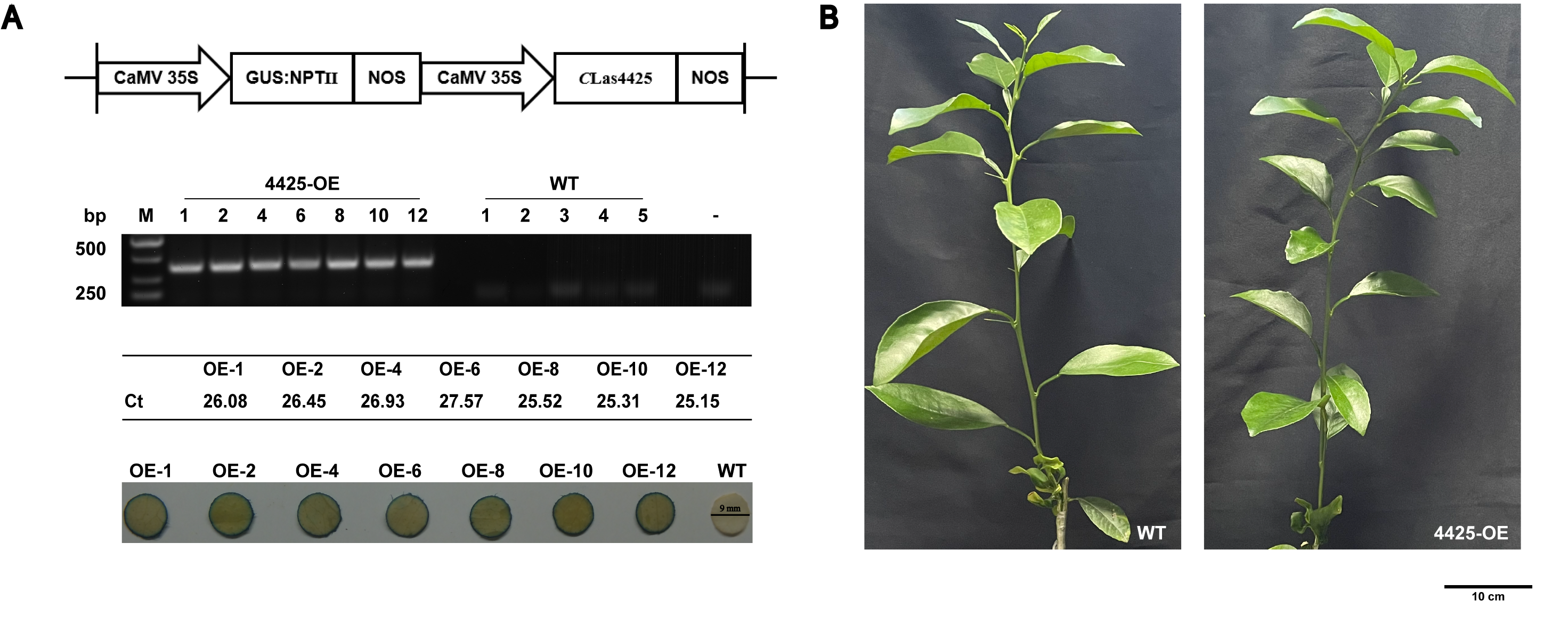

Supplement: Supplementary Figure 4 — (A), Construction of CLas4425-transgenic citrus plants. Structure of the pLGN-CLas4425 plasmid used for the overexpression assays. Identification of transgenic plants with PCR, RT-qPCR, and β-glucuronidase (GUS) histochemical staining. M, DNA marker; OE, transgenic citrus plants; WT, wildtype control. Scale bar: 9 mm. (B), Phenotypes of WT and 4425-OE. Scale bar: 10 cm. [file Image_4.tif]
